# A novel regulation mechanism of the T7 RNA polymerase based expression system improves overproduction and folding of membrane proteins

**DOI:** 10.1038/s41598-018-26668-y

**Published:** 2018-06-05

**Authors:** Federica Angius, Oana Ilioaia, Amira Amrani, Annabelle Suisse, Lindsay Rosset, Amélie Legrand, Abbas Abou-Hamdan, Marc Uzan, Francesca Zito, Bruno Miroux

**Affiliations:** 1 Laboratoire de Biologie Physico-Chimique des Protéines Membranaires, CNRS, University Paris Diderot, Sorbonne Paris Cité, Institut de Biologie Physico-Chimique, Paris, France; 2 Present Address: Helen L. and Martin S. Kimmel Center at the Skirball Institute for Biomolecular Medicine and Department of Cell Biology, NYU School of Medicine, New York, USA; 3 Present Address: Institut de Biologie Intégrative de la Cellule, CNRS, Gif sur Yvette, France

## Abstract

Membrane protein (MP) overproduction is one of the major bottlenecks in structural genomics and biotechnology. Despite the emergence of eukaryotic expression systems, bacteria remain a cost effective and powerful tool for protein production. The T7 RNA polymerase (T7RNAP)-based expression system is a successful and efficient expression system, which achieves high-level production of proteins. However some foreign MPs require a fine-tuning of their expression to minimize the toxicity associated with their production. Here we report a novel regulation mechanism for the T7 expression system. We have isolated two bacterial hosts, namely C44(DE3) and C45(DE3), harboring a stop codon in the T7RNAP gene, whose translation is under the control of the basal nonsense suppressive activity of the BL21(DE3) host. Evaluation of hosts with superfolder green fluorescent protein (sfGFP) revealed an unprecedented tighter control of transgene expression with a marked accumulation of the recombinant protein during stationary phase. Analysis of a collection of twenty MP fused to GFP showed an improved production yield and quality of several bacterial MPs and of one human monotopic MP. These mutant hosts are complementary to the other existing T7 hosts and will increase the versatility of the T7 expression system.

## Introduction

Membrane proteins (MP) represent about 50% of drug discovery targets^1,2^. Despite their importance, structural and biochemical investigations of MP are limited by the difficulty to produce and purify them in a functional state. Half of the unique MP structures present in the Protein Data Bank (PDB) have been obtained after production in *E. coli*, showing that it remains a powerful vehicle in structural biology of MP. The T7 and arabinose promoters have contributed to 80% of all unique MP structures (63% and 17%, respectively)^3^. The T7 RNA polymerase (T7RNAP)-based expression system was designed to maximize the production levels of the target protein^4^. Bacteriophage lambda was modified by inserting the T7 RNA polymerase gene under the control of the *lac L8 UV5* promoter, a variant of the lactose promoter that is insensitive to catabolic repression. Upon addition of IPTG, the T7RNAP enzyme is produced at high levels and subsequently transcribes the target gene which is cloned downstream of a T7 promoter in the expression plasmid. This expression system is powerful but has two major drawbacks. Firstly, the *lacUV5* promoter is leaky and proportional expression of the target gene is difficult to achieve with increasing concentrations of its non-metabolized inducer IPTG. Secondly, Dong and coworkers have shown that overproduction of the T7RNAP enzyme triggers ribosome destruction and cell death^5^. This intrinsic toxicity of the T7 system was used to isolate C41(DE3) and C43(DE3) mutant hosts^6^. Under induction conditions, the level of target mRNA is ten times lower in C41(DE3) than in the parental strain, leading to a slower accumulation of the target protein. Accumulation of the target protein is further delayed in C43(DE3). At the molecular level, Wagner and colleagues showed that in C41(DE3) and C43(DE3), the sequence of the *lacUV5* promoter upstream of the T7RNAP gene in the genome of lambda DE3, reversed to wild-type. However, the L8 mutation in the CRP-binding site was maintained in both strains. This mutation considerably weakens the lac promoter as it cannot be activated by the CRP-cyclic AMP complex^7^. An additional mutation of the *lacI* gene was identified in C43(DE3), which resulted in an amino acid change from valine to phenylalanine at residue 192^8^. The *lacI* gene codes for the *lac* operon repressor, an allosteric protein that binds allolactose or IPTG. This mutation decreased the binding of inducer to the repressor and conferred a super repressor I^s^ phenotype^9^, which further inhibits the transcription initiation of the T7RNAP gene. Given that both mutant hosts have had a major impact on the field of MP structural biology (19% of non *E. coli* membrane protein structures were produced in these T7 expression hosts - 50% of which were complex α-helical MP structures^10^), we aimed at isolating more efficiently regulated hosts with a lower basal level of target gene expression. To achieve this goal, we used the green fluorescent protein (GFP) as reporter gene and we isolated two mutant hosts, C44(DE3) and C45(DE3). By identifying sequence changes in the C44(DE3) and C45(DE3) genomes, we described a radically new T7RNAP regulation mechanism which is based on the nonsense suppressive activity of the BL21(DE3) host. Both C44(DE3) and C45(DE3) mutant hosts extend the promoter strength coverage of the T7RNAP expression system.

## Results and Discussion

### Isolation and characterization of BL21(DE3) derivatives

**Figure 1:**
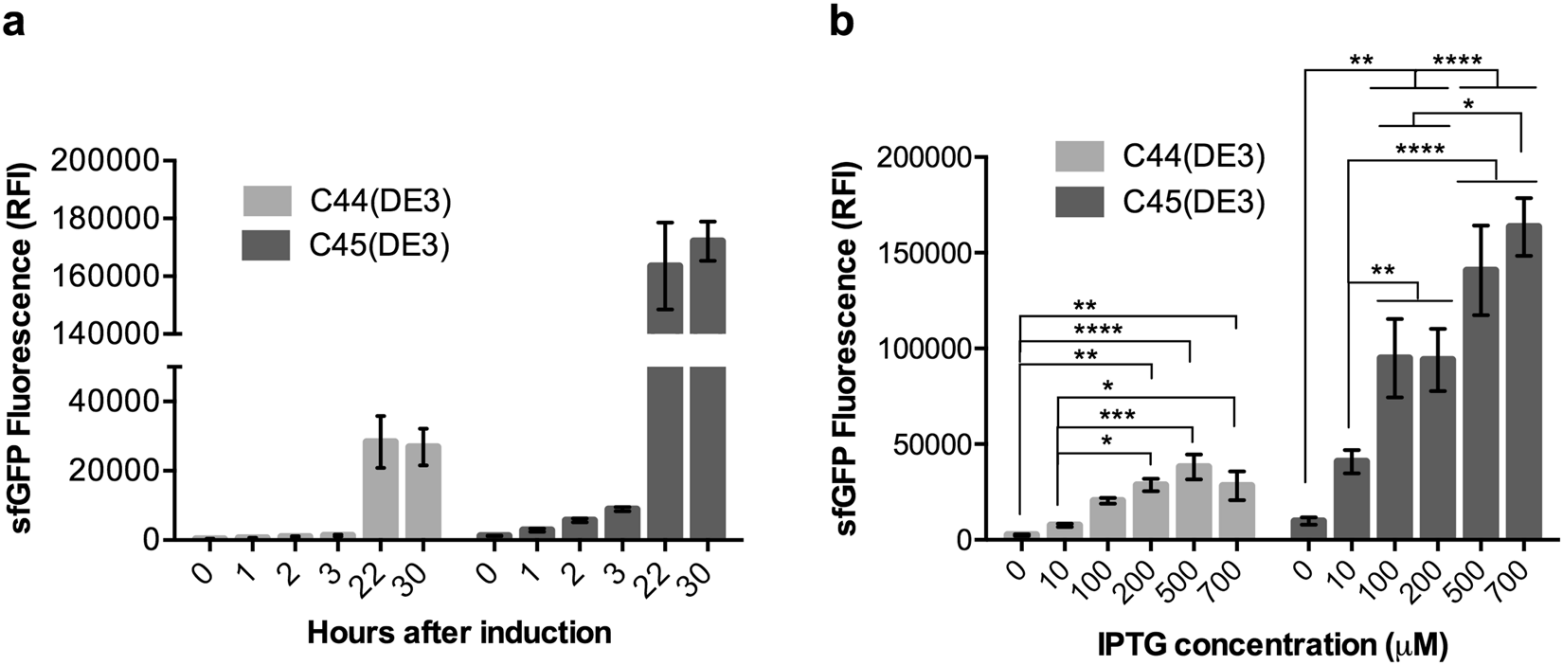
Regulation of the T7 expression system in C44(DE3) and C45(DE3) bacterial hosts. Cells were analyzed by flow cytometry for sfGFP RFI. (**a**) Time course experiment after addition of 0.7 mM IPTG (**b**) IPTG titration experiment. Cells were induced with increasing concentration of IPTG and analyzed after overnight induction. Each plot shows a mean value of three independent experiments. Standard error of the mean is indicated. Statistical significance was analyzed using One-way ANOVA test with Tukey post-hoc correction. F values are: F(5,30) = 9.907 for C44(DE3) and F(5,30) = 19.06 for C45(DE3).

**Figure 2:**
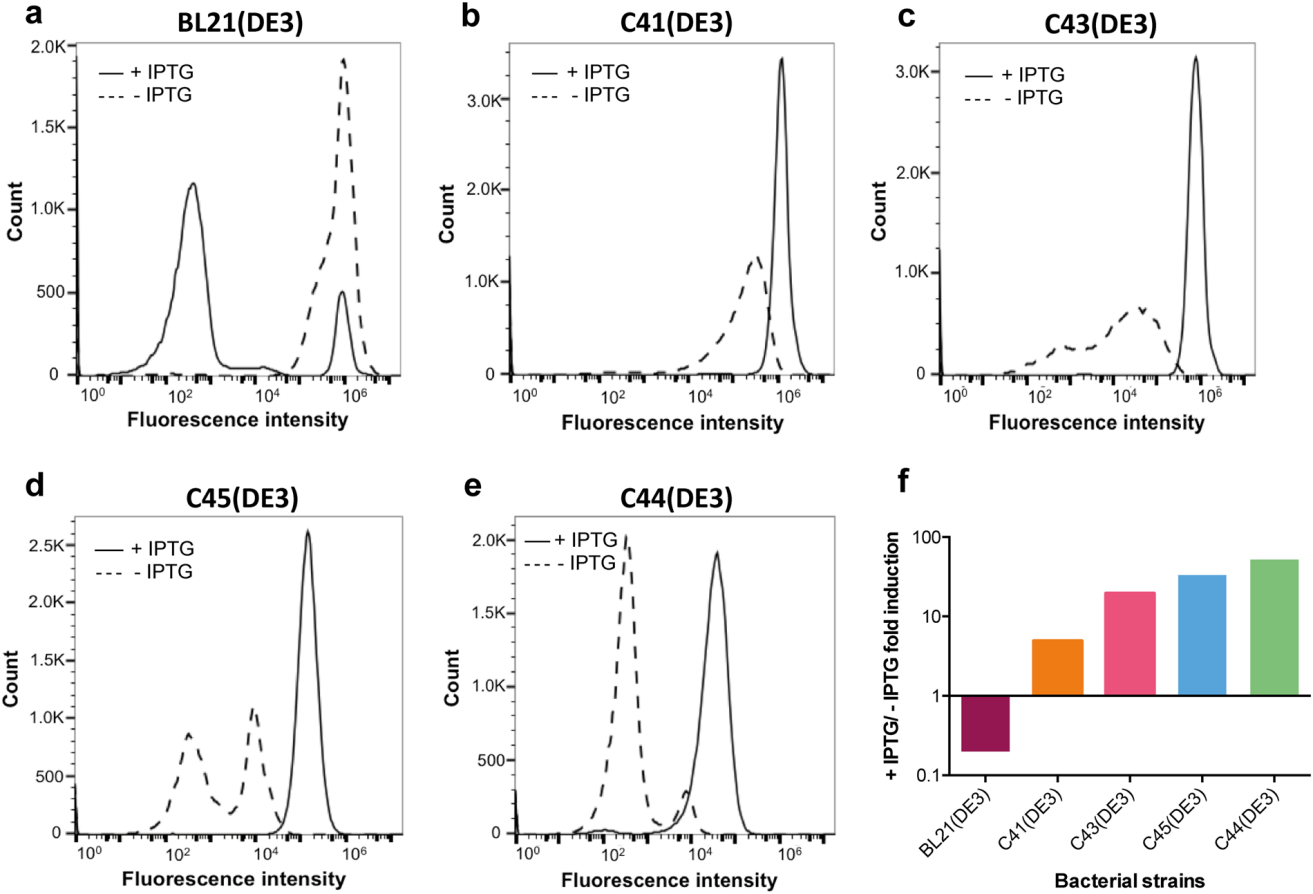
Assessment of the T7 expression hosts using superfolder GFP. Flow cytometry analysis of an overnight culture of BL21(DE3) (**a**) C41(DE3) (**b**) C43(DE3) (**c**) C45(DE3) (**d**) and C44(DE3) (**e**) transformed with pHis17-sfGFP vector. Dashed line indicates RFI of non-induced cells while continuous line shows the RFI of cells induced at OD600 = 0.4 with 0.7 mM IPTG. (**f**) RFI mean value ratio of induced over non-induced cells.

The bacterial mutants C44(DE3) and C45(DE3) were isolated from two independent experiments as described in the materials and methods section. The relative fluorescent intensity (RFI) of sfGFP^11^ was measured by flow cytometry to assess the regulation of the T7 system in all BL21(DE3) derivatives. Three hours after IPTG addition, sfGFP RFI increased by 4 and 7-fold in C44(DE3) and C45(DE3), respectively (Fig. 1a). A subsequent 22 and 18-fold increase of RFI in C44(DE3) and C45(DE3) respectively were observed between 3 h and 22 h after induction. This suggests that the recombinant protein (which in the case of superfolder sfGFP is well reflected by the measured RFI^11^) continues to accumulate in the stationary phase. Both mutant hosts were compared to C41(DE3) and C43(DE3). Fig. S1 shows that the initial rate of sfGFP production is the highest in C41(DE3) and already decreased in C43(DE3), as previously described^6^. In C44(DE3) and C45(DE3) hosts, the rate of sfGFP production is further decreased. In contrast to BL21(DE3), which is difficult to titrate with IPTG (Fig. S2), both C44(DE3) and C45(DE3) could be titrated over an unprecedented range of IPTG concentration starting from 10 μM and up to 0.7 mM (Fig. 1b). Analysis of cell populations showed that in BL21(DE3), the highest RFI was obtained without added IPTG (Fig. 2a) as previously reported^12^. Addition of IPTG to the culture was toxic, as reflected by the appearance of a large number of cells (81% of the bacterial population) that have lost sfGFP fluorescence (Table S1). In contrast, the basal level of sfGFP fluorescence was decreased by a factor of 3.5 in C41(DE3) and addition of IPTG resulted in a homogeneous population of cells (93%) exhibiting the highest level of sfGFP RFI (Fig. 2b and Table S1). In C43(DE3), two populations were observed in the absence of IPTG with a global sfGFP mean RFI of 36,615 + /− 346 (Table S1). Addition of IPTG resulted in a single fluorescent population showing that all cells had kept the ability to produce sfGFP (Fig. 2c). The most remarkable feature of both C44(DE3) and C45(DE3) mutant hosts was their extremely low levels of sfGFP RFI after overnight culture in the absence of IPTG (Fig. 2d,e). For C44(DE3), 89% of the cell population exhibited a sfGFP RFI of 359, which is only 50% above the fluorescence of sfGFP expression plasmid in the absence of T7RNAP (See BL21 RFI in Table S1). Consequently, the induction fold change of sfGFP RFI estimated by flow cytometry increased up to 48 times in C44(DE3) and 33 times in C45(DE3) after overnight induction with IPTG (Fig. 2f). To summarize, the two bacterial expression hosts isolated in this study contain three important features that were not present in any of the existing T7 expression bacterial hosts: *(i)* tight repression of the target gene in high-density culture medium at 37 °C; *(ii)* a tunable level of target protein expression with increasing IPTG concentration; *(iii)* a slow and continuous target protein accumulation in both the exponential and stationary phases at 37 °C. This is achieved without any additional plasmid encoding lysozyme to inhibit the T7RNAP as in the Lemo21(DE3) strain^13^ and without decreasing the growth temperature. The newly isolated hosts offer an unprecedented control of the target gene production levels and, together with C41(DE3) and C43(DE3) hosts, they allow to produce the target gene either at low levels (Table S1, GFP RFI in induced C44(DE3) host) or up to 30 times above the maximum production levels of sfGFP in C44(DE3) (Table S1, induced C41(DE3) host). Differential regulation between T7 hosts adds versatility to the T7 expression system, offering the possibility to either produce the target protein rapidly in exponential phase or slowly during stationary phase. This could be useful for application in synthetic biology, either for the implementation in *E. coli* of complex metabolic pathways or for production of metabolites such as fatty acids^14^.

### Validation of C44(DE3) and C45(DE3) for MP production

**Figure 3:**
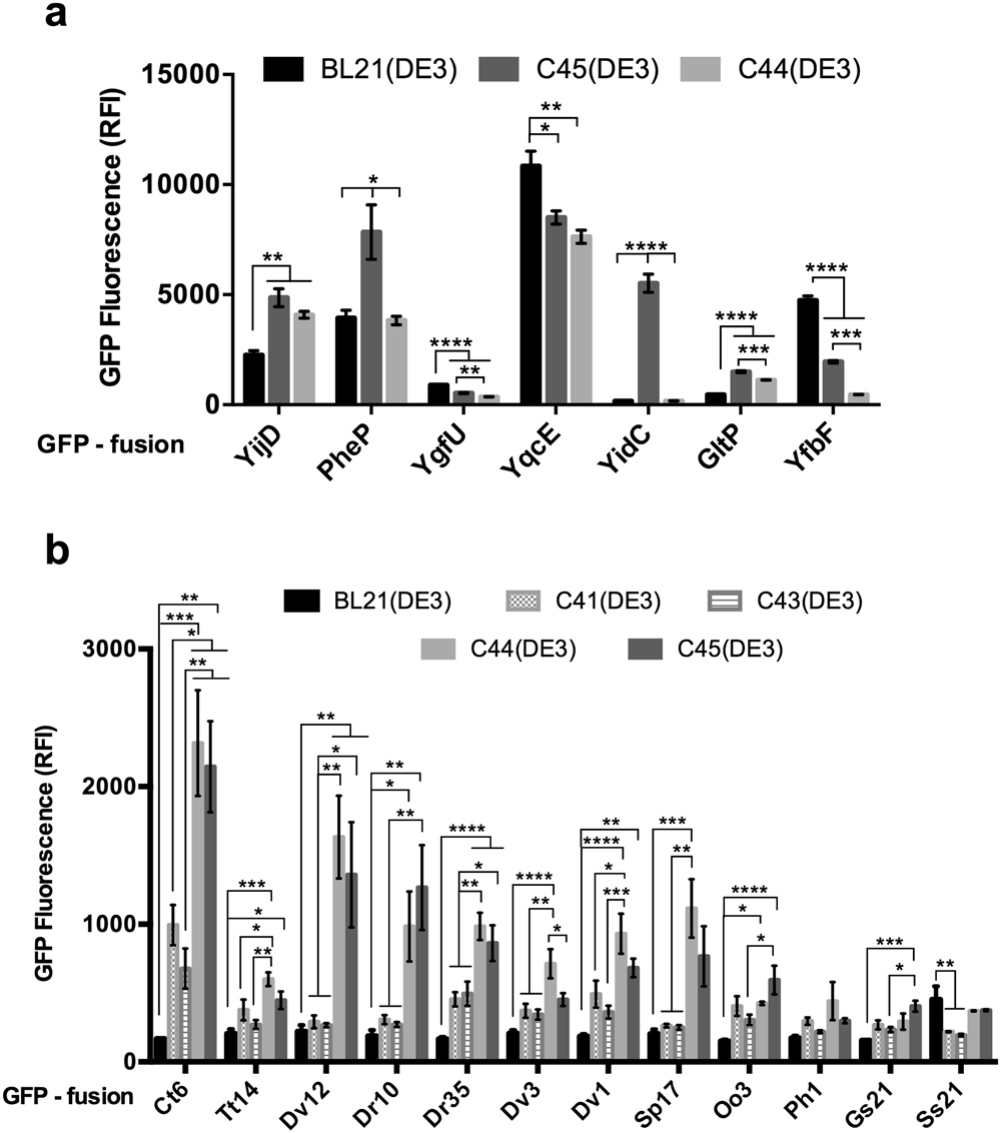
Flow cytometry analysis of MP-GFP production levels. BL21(DE3) cells were grown overnight in LB medium at 30 °C. All other BL21(DE3)-derived hosts were grown in in 2xYT medium, induced at OD600 = 0.4 with 0.7 mM IPTG and further grown overnight at 37 °C. GFP RFI was recorded as described in materials and methods. (**a**) Triplicate cultures producing *E.coli* MP-GFP fusions^16^. (**b**) Six-time replica cultures producing non *E. coli* MP-GFP^19^. Standard error of the mean is indicated. Statistical significance was analyzed using One-way ANOVA test with Tukey post-hoc correction. All F-values and P-values are listed in Supplementary Table S8.

**Figure 4:**
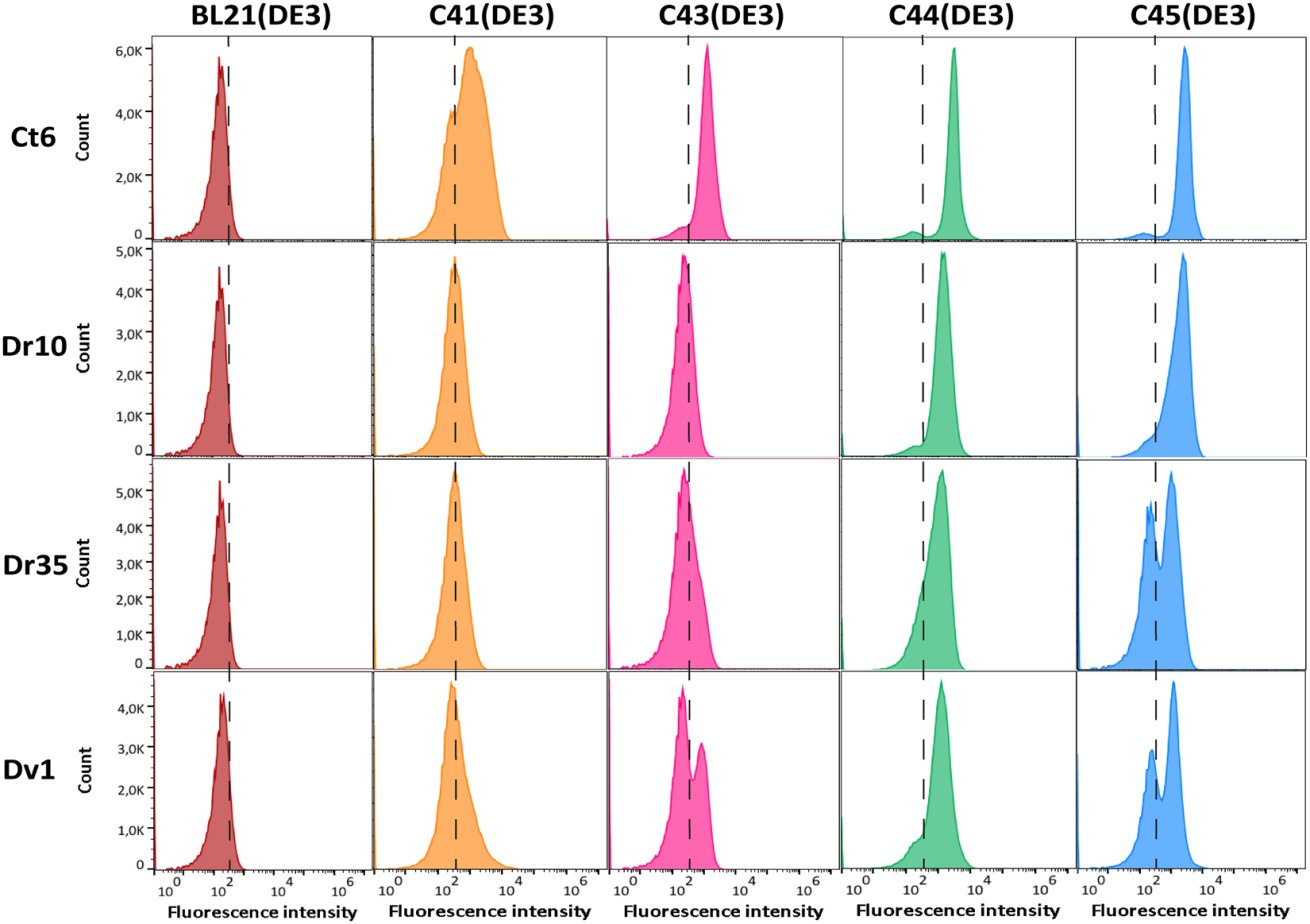
Cell population analysis of four non-*E.coli* MP by flow cytometry. Experimental conditions are described in Fig. 3. The populations of cells producing Ct6-GFP, Dr10-GFP, Dr35-GFP and Dv1-GFP in BL21(DE3) and BL21(DE3)-derived hosts were analysed by flow cytometry based on GFP fluorescence. The dashed line marks the autofluorescence of bacteria.

**Figure 5:**
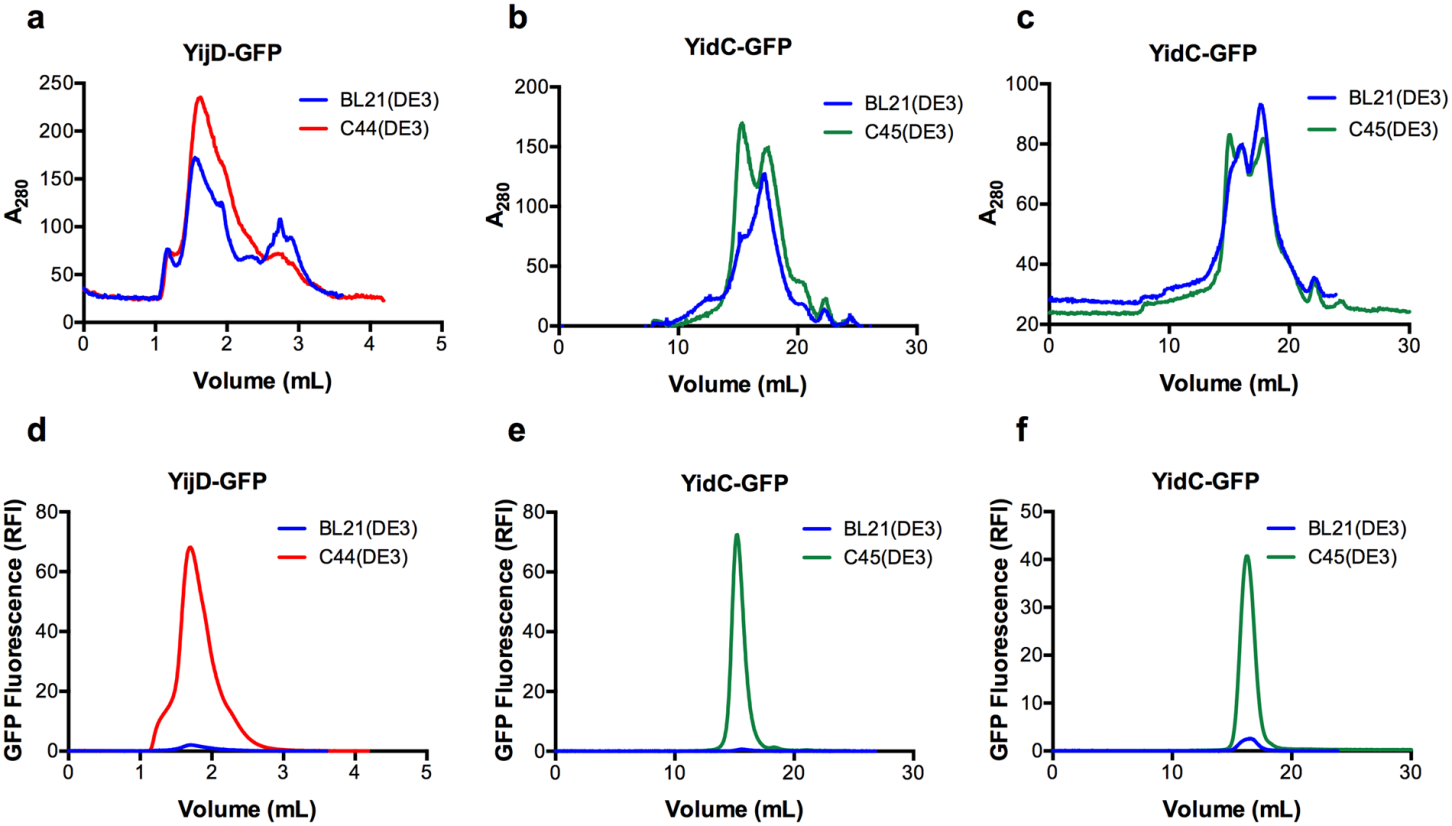
Quantification of MP-GFP production levels by Fluorescence detection size-exclusion chromatography. BL21(DE3), C44(DE3) and C45(DE3) host strains were transformed either with YijD-GFP (**a**,**d**) or YidC-GFP (**b**,**c**,**e**,**f**) expression vectors. Cell pellets from 200 ml culture (**a**,**b**,**d**,**e**) or a fix amount of membranes (**c**,**f**, 18 mg at 2 mg/mL) were solubilized with DDM (1% final concentration) and 100 μl of solubilized material was injected on gel filtration column. Absorbance at 280 nm (**a**–**c**) was recorded while GFP fluorescence (**d**–**f**) was analyzed using a Jasco detector.

C44(DE3) and C45(DE3) hosts are very well repressed before induction but this is at the expense of the induction power. Indeed sfGFP levels in those mutant hosts are about ten and seven times lower than in C41(DE3) and C43(DE3) respectively (Table S1). T7 expression hosts have been extensively tested for MP production in different culture conditions. Lee and coworkers showed that culture media, inducer concentrations, especially for the Lemo21(DE3) host^13^, strongly influence the final yields of the target MP. For C43(DE3) host, LB medium resulted in low yields while maximum levels of 10 model MPs were obtained either in TB or PASM-5052 media when cells were induced with 0.4 mM IPTG at 25 °C^15^. For BL21(DE3) host, Zhang *et al*. have recently shown that omitting IPTG in a LB culture grown at 30 °C was the optimum condition to achieve high levels production of a set of 10 *E. coli* MPs^12^. This result is consistent with our comparative analysis with sfGFP (Table S1). Decreasing the temperature had no effect on the expression level of the target MP in C44(DE3) and C45(DE3) (data not shown), therefore we tested GFP-MP fusions in the simplest and cheapest condition: 37 °C, 2xYT medium and 0.7 mM IPTG induction at 0.4 OD_600nm_. For BL21(DE3) host, expression conditions described in Zhang *et al*. were used. Two collections of expression plasmids fused to GFP and encoding MP from *E. coli* and other bacterial species (MP-GFP, see Table S2) were tested. Firstly, the toxicity associated with the overproduction of the MP-GFP fusion was assessed on solid medium. For each construct, cells were diluted 10^7^-fold and were plated on 2xYT agar medium with or without IPTG. The surface area (SA) of colonies was measured from photographic pictures using Image J software. The relative colony surface area (RCSA) upon induction was calculated (SA(+IPTG)/SA(-IPTG)*100) and is presented in Table S3. In the BL21(DE3) host, all expression vectors, including the soluble protein sfGFP, prevented colony formation on 2xYT plates supplemented with 0.7 mM IPTG (RCSA = 0, Table S3). In contrast, all MP-GFP fusions allowed colony formation on IPTG containing plates in either C44(DE3) or C45(DE3), with the exception of Ph1-GFP and Gs21-GFP (Table S3). The RCSA values were generally lower in C45(DE3) for most of the MP-GFP fusions as well as for the pHis-sfGFP control plasmid. Next, GFP fluorescence was measured by flow cytometry in liquid cultures. As shown in Fig. 3a, among seven *E. coli* MP-GFP expression plasmids tested in this study, four target proteins, namely YijD, PheP, YidC and GltP were produced at significantly higher levels in either C44(DE3) or C45(DE3) bacterial hosts, while three constructs (YgfU, YfbF, and YqcE) remained better produced in the BL21(DE3) host. To expand our results, twelve non *E. coli* MP-GFP^16^, were produced in BL21(DE3) and BL21(DE3) derivatives. Almost all constructs were produced at significantly higher levels in either C44(DE3) or C45(DE3) bacterial hosts (Fig. 3b). Cell population analysis is presented in Fig. 4 with four GFP-MP (Dr10, Dr35, Ct6, and Dv1) that are representative of the phenotypes observed between bacterial hosts. For Dr10-GFP and Ct6-GFP expression plasmids, C44(DE3) and C45(DE3) mutant hosts exhibited a homogenous expression pattern as reflected by the narrow distribution of GFP positive cells within one order of magnitude of RFI. In contrast, expression pattern of both GFP-MP in C41(DE3) and C43(DE3) was extended over two order of magnitude of RFI due to the presence of cells that have lost the ability to produce the target MP-GFP. Expression of Dr35-GFP and Dv1-GFP resulted in a nearly homogenous expression pattern only in C44(DE3) while negative cells were detected in C45(DE3) and in much larger amount in C43(DE3) and C41(DE3) hosts. Altogether these results show that one of the main advantage of C44(DE3) and C45(DE3) hosts relies in the homogenous expression pattern of the target gene, with few negative cells, which could explain why the mean GFP RFI was increased in those examples. Whole cell GFP fluorescence analysis can be misleading due to possible GFP cleavage from the MP and does not reflect the exact fraction of MP that can be extracted from bacterial^17^ or yeast^18^ membranes. In order to assess more precisely the quality and quantity of MP-GFP fusions produced, we used the Fluorescence-detection size-exclusion chromatography (F-sec) method^19^. Two *E.coli* proteins, namely YijD and YidC were produced in 200 mL volume cultures; equal volume of membrane preparations were solubilized in presence of 1% DDM and subjected to F-sec analysis as described in materials and methods. Figure 5 shows that, per volume of culture, YijD-GFP RFI increased 34-fold when extracted from C44(DE3) membranes (Fig. 5d), while YidC-GFP RFI increased 105-fold in C45(DE3) solubilized membranes (Fig. 5e). To solubilize MP from both bacterial hosts with the same detergent-protein ratio, we repeated the experiment with YidC-GFP with a fixed amount of proteins (18 mg with 1% DDM in 9 mL final volume). The F-sec analysis showed that the fusion protein level increased 20-fold in C45(DE3) bacterial membranes (Fig. 5f). Since the total amount of MP obtained from a 1 L culture of C45(DE3) cells quadrupled (126 mg in C45(DE3) versus 32 mg in BL21(DE3)), the yield per liter of culture of YidC-GFP increased 80-fold, a fold change close to the one measured per volume in Fig. 5e.

**Figure 6:**
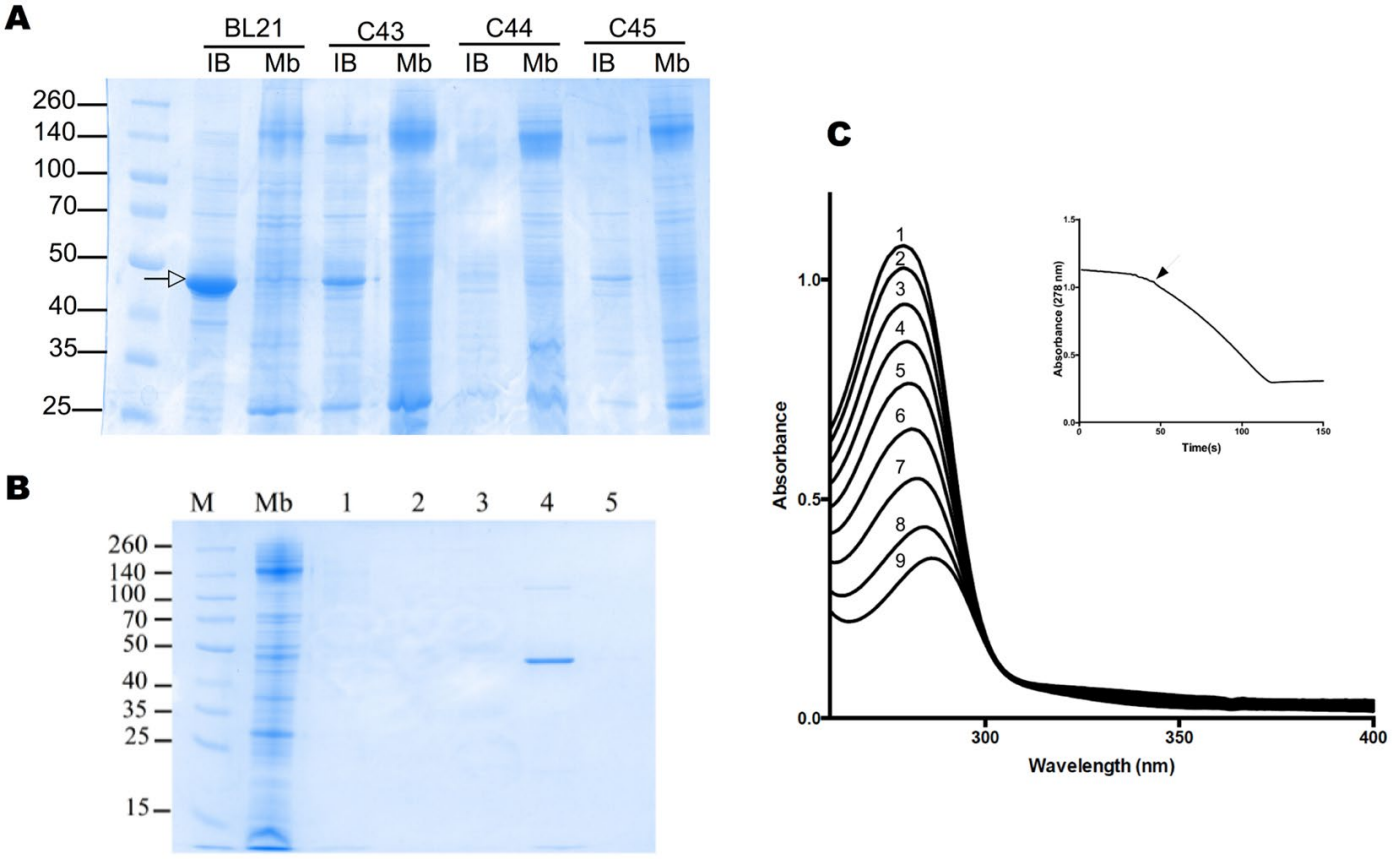
Functional expression of recombinant h-SQR in C45(DE3). (**A**) SDS-PAGE analysis of h-SQR inclusion bodies formation in BL12(DE3) (arrow) and BL21(DE3) derivatives. 30 μg of inclusion bodies (IB) or membrane fraction (Mb) proteins were analyzed by SDS-PAGE stained with Coomassie Blue solution. Lane M: molecular weight marker. (**B**) SDS-PAGE analysis of h-SQR purification. Membrane fraction (Mb), flow-through (1) column wash with 20 mM (2) and 40 mM (3) Imidazole, column elution with 200 mM (4) and 500 mM (5) Imidazole. (**C**) Spectral course of h-SQR catalytic assays. The reaction mixture contained 100 μM CoQ1 and 400 μM of Na2 S. The activity of 0.2 μg h-SQR was recorded during 600 s at 287 nm. Curves 1 to 9 were recorded every 10 s after h-SQR addition. The inset shows the time course of absorbance changes at 287 nm, black arrow show h-SQR addition.

In order to test if C44(DE3) and C45(DE3) mutant hosts could expand the sequence coverage of MP to eukaryotic proteins, we tested the human sulfide quinone oxydo-reductase (h-SQR). h-SQR is a monotopic MP that has been previously produced in *E. coli* at moderate levels (250 μg/L of culture of cells grown overnight at 15 °C) only in presence of the companion plasmid pCPN10/60, which produces cold-adapted chaperon proteins from *Oleispira Antarctica*^20^. The same synthetic gene used in^20^ encoding for the h-SQR was introduced in pHis17 and transformed either in BL21(DE3) or in the four other mutant hosts. The recombinant protein was produced both at the *E. coli* membrane and aggregate as inclusion bodies in all bacterial hosts. However inclusions body levels were the lowest in C44(DE3) and C45(DE3) (Fig. 6A). To confirm that the protein was correctly folded in C45(DE3) bacterial membranes, h-SQR was purified from a 1 L culture (Fig. 6B) with a final yield of 600 μg of purified protein/L (twice the yield obtained in^20^) and assayed for activity using Na_2_S as a substrate. The resulting activity curves (Fig. 6C) were similar to those previously published^20^, and confirmed that the protein is correctly folded, binds its cofactor FAD, and is able to oxidize Na_2_S (Km = 11 mM). Membrane protein production often induces a stress to the host cell. In some cases it has been shown that the target membrane protein is by itself toxic to the cell^21^. In most cases, however, the T7 expression system overloads the translocation and membrane insertion machineries very early after induction, causing an excess of the unfolded target protein that aggregates rapidly as inclusion bodies^22^. Heat-shock protein, unfolded stress response, and proteases often appear as a desperate attempt to circumvent this problem (for review see^23^). Wagner and colleagues have nicely illustrated this view by conducting a global proteomic study with YidC as a model protein partially produced as inclusion bodies^24^. They show that overproduction of the YidC membrane protein critically affects BL21(DE3)pLysS cell growth and increases chaperone (DNAJ/K, GROEL/S) and protease (HslU/V, ClpXP, ClpB) levels. Interestingly, the same authors found that inclusion bodies formation was greatly decreased in C41(DE3) or C43(DE3) mutant hosts, and that toxicity in the form of chaperone and protease induction was strongly reduced^13^. In C45(DE3) host, toxicity linked to YidC production was low and comparable to the one observed with the soluble protein sfGFP (Table S3) and solubilized YidC levels increased 105 times in C45(DE3) membranes as compared to the BL21(DE3) host (Fig. 5). Similarly, we have shown that the h-SQR is readily targeted to the bacterial membrane in C45(DE3) without the need of chaperones or of growing cells at 15 °C. From these experiments it appears that, despite of its weak induction power seen with sfGFP, C45(DE3) host is still suitable for MP production. When cells are grown overnight at 37 °C in 2xYT medium, it surpasses all T7 hosts tested so far for producing and targeting foreign MPs at the *E. coli* membrane. These growth conditions are ideal for *E. coli* physiology and compatible with large scale fermentation at low cost. Having a “weak” host in the “strong” T7 expression system allows to produce the target protein at low to moderate levels and to reduce the risk of misfolding and formation of inclusion bodies. Testing other hosts such as C41(DE3) and C43(DE3) or Lemo21(DE3), which are able to produce the target gene at much higher levels (See Lee *et al*. and sfGFP expression levels in Table S1) but with the risk of inclusion bodies formation, can still be useful. In some cases growing cells at lower temperature overcomes the formation of inclusion bodies. As a matter of fact, the highest levels of YidC protein are achieved in C43(DE3) host induced at 25 °C (data not shown) but expression of the h-SQR still produces inclusion bodies in this milder condition. Therefore C41-5(DE3) hosts form a complementary set of hosts to screen, with the same expression plasmids, different strengths of induction and different kinetics of target gene accumulation.

### Analysis of the T7 gene regulation in C44(DE3) and C45(DE3)

**Figure 7:**
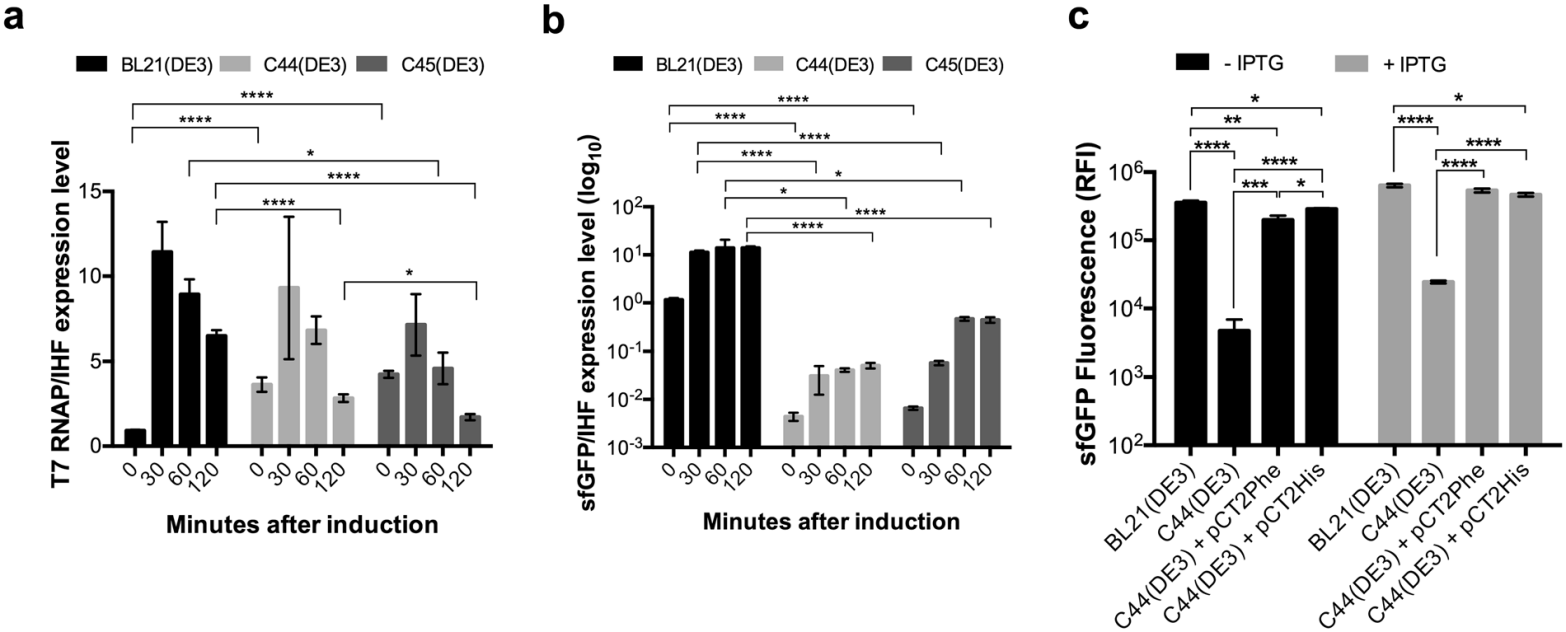
Translational regulation of the T7RNAP gene in C44(DE3) and C45(DE3) hosts. RT-qPCR was performed as described in materials and methods. IHF was chosen as reference gene. (**a**) T7RNAP/IHF expression levels (**b**) sfGFP/IHF expression levels. (**c**) Cells were transformed either with sfGFP as control or with sfGFP and pCT2Phe or pCT2His plasmid. The graph represents sfGFP RFI analyzed by flow cytometry after overnight cultures with (gray columns) or without addition of IPTG (black columns). Three independent experiments were performed with technical triplicate. Standard error of the mean is indicated on each plot. Statistical significance was analyzed using One-way ANOVA test and Tukey post-hoc correction.

In order to identify the mechanisms that govern the induction of the T7RNAP by IPTG in the isolated mutant hosts, the genomes of BL21(DE3) and its derivatives were entirely sequenced as described in materials and methods section. All mutations were confirmed by PCR analysis (see Table S4 for primer list, Fig. S3). Our data (Table S5) confirmed the previously published genome analyses of C41(DE3) and C43(DE3) strains^8,25^. In C44(DE3) we found two mutations (Table S6). The first mutation was positioned in the *rbsD* gene, which had also been identified by others^8,25^ in C41(DE3) and C43(DE3) genomes. In BL21(DE3), the *rbsD* gene encoding D-ribose pyranase is interrupted by the insJK5 insertion. According to Schlegel and co-workers^25^, the presence of an IS-element in *rbsD* prevents efficient growth of BL21(DE3) on ribose as its sole carbon and energy source. Deletion of the insJK5 insertion restores the wild-type *rbsD* gene in C41(DE3), C43(DE3), C44(DE3), and C45(DE3) derivatives, enabling efficient growth on ribose. The second mutation consists of an *amber* stop codon at the position Glu_656_ of the T7RNAP. Interestingly, we found that C45(DE3) also carries an *ochre* stop codon at position Gln_359_ of the T7RNAP, which highlights a common genetic trait between both C44(DE3) and C45(DE3) mutants (Table S6). However, the pattern of genetic changes in C45(DE3) bacterial mutant is more complex because of three additional deletions in *gltL, B-lom* and *ycdX loci*. The T7RNAP enzyme contains three C-terminal domains (A, B and C) that are critical for the catalytic activity of nucleotide polymerases^26^. In C45(DE3) host, the truncated T7RNAP lacks all three C-terminal domains and is therefore most likely inactive. In C44(DE3) host, the T7RNAP enzyme is truncated just after the B catalytic domain. Although C-terminal deletions of this enzyme have not been systematically studied, the C domain is conserved in all nucleotide polymerases and the point mutation Asp_812_Asn at the end of the protein completely abolishes its activity^27^. To demonstrate our hypothesis, T7RNAP and sfGFP mRNA levels were measured by RT-qPCR experiments. Figure 7a shows only moderate reductions in T7RNAP mRNA levels (which cannot account for T7RNAP protein levels falling below its antibody detection levels, data not shown) in C44(DE3) and C45(DE3) as compared to BL21(DE3) host. In contrast, the target sfGFP mRNA levels, controlled by the T7RNAP activity, were dramatically decreased in both mutant hosts (Fig. 7b) as compared to BL21(DE3) host for two reasons. Firstly, the basal levels of sfGFP mRNA, before addition of IPTG, showed a 200–fold decrease in C45(DE3) and a 300-fold decrease in C44(DE3), when compared to BL21(DE3). Secondly, the maximal levels of sfGFP mRNA after IPTG addition showed a 270-fold and 30-fold decrease in C44(DE3) and C45(DE3) respectively. Next, we co-expressed *amber* suppressors in C44(DE3) and tested whether sfGFP expression phenotypes could be reversed to that of the parental strain BL21(DE3). We used the pCT2Phe and pCT2His *amber* suppressor vectors^28^, which mediate the insertion of a phenylalanine or a histidine at the amber stop codon position respectively. C44(DE3) cells transformed with both pHis-sfGFP and pCT2Phe or pCT2His plasmids were unable to form colonies on IPTG containing plates (data not shown). Flow cytometry analysis showed that in C44(DE3) cells expressing *amber* suppressor, sfGFP RFI reached the level of the BL21(DE3) parental host in both overnight induced and non-induced cultures (Fig. 7c). Thus, we were able to fully demonstrate the mechanism of regulation of the T7 system in C44(DE3). The production of the target protein depends on the translation of the full-length T7RNAP enzyme, which is possible only when ribosomes read through the stop codon mutations introduced in the T7RNAP gene. In fact, a very weak suppressive stop codon activity has been incidentally observed in BL21(DE3)^29^, likely accounting for the synthesis of minute amounts of the T7RNAP enzyme from the high levels of T7RNAP mRNA available after IPTG addition. To conclude, The T7RNAP gene in C44(DE3) and C45(DE3) mutant hosts is regulated at transcriptional levels as it is in BL21(DE3). However, the presence of a premature termination codon within the T7RNAP gene of each of these strains produces an inactive truncated polymerase. Expression of the target gene relies only on the very weak basal level of suppression of either amber or ochre stop codon. Thus, the levels of T7 polymerase in C44(DE3) and C45(DE3) are the lowest of all the BL21(DE3) derived T7-expression hosts isolated so far. This probably explains why these strains show different recombinant production phenotypes.

Several groups have developed genetic screens and synthetic biology approaches to explain recombinant protein production in more detail and ultimately to circumvent the associated toxicity^2,30,31^. They identified stress response genes (*ybaB, dnaK, dnaJ, djlA, rraA*) as toxicity suppressors upon MP overproduction. In contrast, the study of Baumgarten and colleagues, who used YidC-GFP fusion to isolate a mutant host in which a mutation in the T7RNAP strongly decreases its binding to the T7 promoter^32^, and our present results, show that fine-tuning of target MP transcription is also a powerful and complementary approach to suppress toxicity and achieve high cell density and high levels of MP production. Together with direct evolution of the target gene^33^ and codon usage adaptation^34–37^, the C44(DE3) and C45(DE3) bacterial hosts will most likely expand the use of *E. coli* for MP production.

## Materials and Methods

### Bacterial strains and plasmids

*Escherichia coli* strains C41(DE3) and C43(DE3) have been previously described^6,38^. All MP used in this study are listed in Table S2. The pHis17 and pMW7-GFP-Xa plasmids were derived from pMW7^39^. The pHis17 plasmid was modified by addition of three 6*HIS-Tag immediately in frame after the BamHI, HindIII and EcoRI cloning restriction sites. The pMW7-GFP-Xa vector contains GFP with a factor Xa cleavage sequence in frame of the C-terminal end of GFP. The gene encoding the sfGFP protein was amplified from the vector pDHL1029^40^ using the forward primer 5′CAGGGATCCAGTAAAGGTGAAGAACTG3′ and the reverse primer 5′CAGAAGCTTTTATTTGTAGAGTTCATC3′ and was subcloned into pHis17. A synthetic optimized version of the gene encoding human sulfide-quinone oxydo-reductase (h-SQR)^20^ was obtained from Eurofins genomics, and subcloned between the BamHI and EcoRI sites of the pHis17 plasmid. Amber suppressor plasmids pCT2Phe and pCT2His (cam, Su(UAG)Phe or His), constructed in pACYC184, are described by Lenfant *et al*.^28^.

### Mutant strains selection

The *E.coli* strains C44(DE3) and C45(DE3) were isolated in two independent selection experiments using pMW7-GFP-Xa as gene reporter^3,41^. Briefly, BL21(DE3) cells were transformed with pMW7-GFP-Xa. A single colony was inoculated in 50 mL 2xYT medium supplemented with ampicillin. IPTG (0.7 mM) was added at OD_600nm_ 0.4. Serial dilutions of the culture were made 2 hours after IPTG addition and plated on 2xYT plates containing IPTG (0.7 mM) and ampicillin. GFP-expressing colonies were revealed under UV light (300 nm UV lamp to avoid additional mutations) and subcloned onto 2xYT ampicillin plates. The next day, only colonies not exhibiting visible green fluorescence were selected for pMW7-GFP-Xa plasmid removal^6^.

### Genome analysis

Chromosomal DNA was prepared with the Wizard^®^ Genomic DNA Purification Kit (Promega, U.S.A.). Whole genome analysis of C44(DE3) and C45(DE3) was performed at the genomic platform of the Pasteur Institute (Paris) using an Illumina Miseq sequencing. Genome sequences were deposited at NCBI (SAMN08011119 and SAMN08011120 for C44(DE3) and C45(DE3) respectively). The reads were mapped onto the reference genome sequence of BL21(DE3) (GenBank ID AM946981).

### Production of the recombinant proteins

The C41(DE3), C43(DE3), C44(DE3) and C45(DE3) bacterial hosts were grown overnight in 2xYT broth supplemented with ampicillin (100 μg/mL) when harboring the pHis17 or pMW7 plasmids or with kanamycin (50 μg/mL) for all the other plasmids. Overnight pre-cultures were diluted 1:100 and cultures were grown at 37 °C in an Infors HT Minitron at 130 rpm. For all bacterial hosts except for BL21(DE3), protein production was induced by adding 0.7 mM IPTG to the media when the culture had reached an OD_600_ of 0.4. The BL21(DE3) host harboring either pET28a + or pLIC expression plasmids was cultivated overnight without addition of IPTG according to Zhang and coworkers^12^ to optimize the protein production in Luria-Bertani (LB) broth at 30 °C. For the human SQR, temperature of growth was lowered to 30 °C for the BL21(DE3), C44(DE3), and C45(DE3) strains, and to 25 °C for C43(DE3). Cells were harvested by centrifugation (2500 × *g* for 10 min.) and cell pellet was suspended in 30 mL of 50 mM Tris-HCl buffer, pH 7.4 supplemented with 1 × protease inhibitor cocktail (Thermo scientific). The cells were lysed using the Cell Distruptor at a pressure of 2 kBar (Constant Systems LTD). Cell debris were removed by centrifugation at 2,500 × *g* for 10 min. Inclusion bodies were recovered at 10,000 × *g* for 20 min. Membranes were recovered after centrifugation at 100,000 × *g* of the low speed supernatant for 1 h.

### Flow cytometry

After overnight induction, the cells were washed with PBS (10 mM Phosphate, 150 mM NaCl, pH 7.4) and diluted to an OD_600 nm_ of 0.01. They were analyzed and counted on a C6 Accury flow cytometer with an acquisition threshold of 10,000 on forward scatter (FSC) and of 3,000 on side scatter (SSC) to remove electronic noise and small contaminants. Their fluorescence was measured on a FL1 detector (emission detection 533/±30 nm) with forward and side scatter values above 20,000 and 12,000 respectively. With this setting, BL21 cells had a mean relative autofluorescence intensity around 200. The mean fluorescence value of the whole population was chosen as a global indicator of the expression level of the recombinant protein in each bacterial host culture.

### Purification and activity assay of the h-SQR

All purification steps were conducted at 4 °C. The membrane fractions were diluted at 2 mg/mL protein concentration in 50 mM Tris-HCl buffer (pH 7.4), 50 mM NaCl, 10% glycerol, 1 × protease inhibitor cocktail and solubilized in presence of 2% DDM for 1 h, at 4 °C. Insolubilized material was removed by centrifugation at 100,000 × *g* at 4 °C for 1 hour. The high speed supernatant was collected and diluted in 100 mL final volume of buffer A (100 mM Tris-HCl, pH 8, 150 mM NaCl, 10% glycerol) with 5 mM Imidazole. The sample was loaded onto a His Pur^TM^ Ni-NTA Chromatography column (Thermo Scientific) equilibrated with buffer A with 5 mM Imidazole and 0.05% DDM. After 10 volumes column washes with buffer A containing 20 mM and 40 mM Imidazole and 0.05% DDM, h-SQR was eluted with buffer A containing 0.05% DDM and 200 mM Imidazole. Activity assays were conducted under anaerobic conditions at room temperature using a cuvette with a screw-cap equipped with a Teflon-silicon septum. The sulfide-quinone oxidoreductase activity was measured by monitoring the reduction of Coenzyme Q_1_ at 278 nm according to^20^. The CoQ_1_ reduction rates were determined after subtraction of the initial slope obtained before addition of h-SQR. The CoQ_1_ absorption at 278 nm was corrected at 700 nm.

### Fluorescence-detection size-exclusion chromatography

Bacterial membranes of 200 mL volume culture were resuspended in 5 mL solubilization buffer (20 mM Tris-Hcl, pH 8, 150 mM NaCl and 1% DDM). After one hour solubilization at 4 °C, insoluble material was discarded by ultracentrifugation for 30 min at 100,000 × *g*. To avoid different protein/detergent ratios between different strains, 18 mg (2 mg/mL) of membranes were solubilized with 1% DDM in 20 mM Tris-Hcl, pH 8.0, 150 mM NaCl buffer for 1 h at 4 °C. Solubilized MP (100 μL) were loaded on a Superose-6 10/300GL column (GE-Healthcare) for YidC-GFP or onto a Superdex-200 5/150GL column (GE-Healthcare) for YijD-GFP. Size-exclusion chromatography was performed in presence of TBS supplemented with 0.03% DDM. Fluorescence detector (FP-2020, Jasco) was used to measure GFP relative fluorescent intensity with an excitation wavelength of 488 nm and emission wavelength of 512 nm.

### Real time quantitative PCR gene expression analysis

Total RNAs were extracted from cells using a phenol-chloroform protocol^3^. The cDNA synthesis was performed using the QuantiTect reverse transcription Kit (Qiagen) according to manufacturer’s protocol. RT-qPCR was carried out in triplicate using the SsoAdvanced™ Universal SYBR^®^ Green Supermix (Biorad) and the CFX96 Touch™ Real-Time PCR Detection System (Biorad). IHF mRNA served as internal control for standardization. Primers used are listed in Table S7.

### Statistical analyses

All the experiments, unless specified, were made in triplicate. Error bars were calculated taking into account the standard error of the mean (SEM). The null hypothesis, i.e. are expression levels of each recombinant MP different between two expression hosts?, was tested by using the one-way ANOVA with a post-hoc Tukey test using PRISM program. P values < 0.05 were considered statistically significant. Stars in figure indicate the order of magnitude of the P values: *p value < 0.05; **p < 0.01; ***p < 0.001; ****p < 0.0001. All F-values and P-values are listed in Supplementary Table S8.

## Electronic supplementary material

Supplementary Informations
