# Supplementary material for: A novel regulation mechanism of the T7 RNA polymerase based expression system improves overproduction and folding of membrane proteins
Source: Sci Rep. 2018 Jun 5;8:8572. doi: 10.1038/s41598-018-26668-y (PMC5988807; doi:10.1038/s41598-018-26668-y)
Supplement: Supplementary file 1 — Supplementary Informations [file 41598_2018_26668_MOESM1_ESM.pdf]

A novel regulation mechanism of the T7 RNA polymerase based expression system improves overproduction and folding of membrane proteins

Federica Angius[1], Oana Ilioaia[1], Amira Amrani[1], Annabelle Suisse[1,2], Lindsay Rosset[1], Amélie Legrand[1], Abbas Abou-Hamdan[1,3], Marc Uzan[1], Francesca Zito[1] and Bruno Miroux[1]

[1] Laboratoire de Biologie Physico-Chimique des Protéines Membranaires, Institut de Biologie Physico-Chimique, CNRS, UMR7099, University Paris Diderot, Sorbonne Paris Cité, Paris, France

[2] Present address: Helen L. and Martin S. Kimmel Center at the Skirball Institute for Biomolecular Medicine and Department of Cell Biology, NYU School of Medicine, New York, USA

[3] Present address: Institut de Biologie Intégrative de la Cellule, CNRS, Gif sur Yvette, France

Correspondence address :

Bruno.Miroux@ibpc.fr

Laboratoire de Biologie Physico-Chimique des Protéines Membranaires, UMR7099-CNRS IBPC, 13 rue Pierre et Marie Curie 75005 Paris, France

Telephone: 33 1 58 41 52 25

Fax: 33 1 58 41 52 24

Keywords:

production of membrane proteins, *E. coli*, T7 RNA polymerase

Table S1: analysis of the sfGFP fluorescent intensity in expression hosts

Bacterial host	Induction	Total mean RFI[1] (SEM[2])	RFI mean (% population)	
			Low[3]	High[4]
BL21	+IPTG	296 (16)	216(98)	4021(2)
BL21	-IPTG	278(27)	244(99)	2836(1)
BL21(DE3)	+IPTG	184,728 (29,173)	869 (81)	965,333 (19)
BL21(DE3)	-IPTG	788,656 (35,374)	368 (5)	832,333 (95)
C41(DE3)	+IPTG	1,102,447 (24,083)	5,153 (7)	1,186,667 (93)
C41(DE3)	-IPTG	223,500 (10,838)	434 (6)	238,935 (94)
C43(DE3)	+IPTG	713,016 (27,837)	980 (6)	754,666 (94)
C43(DE3)	-IPTG	36,615 (346)	792 (32)	53,394 (68)
C44(DE3)	+IPTG	60,988 (23,242)	130 (3)	37,979 (97)
C44(DE3)	-IPTG	1,268 (52)	359 (89)	7,688 (11)
C45(DE3)	+IPTG	157,115 (5,239)	262 (2)	159,361 (98)
C45(DE3)	-IPTG	4,717 (385)	468 (60)	11,082 (40)

[1] RFI, relative fluorescence intensity after overnight induction [2]SEM standard error of the mean in three independent experiments; [3]Low RFI population, [4]High RFI population.

Table S2. Membrane proteins used in this study

Protein name	Origin	Molecular weight/TM	Fusion partner	UniprotKB number
YidC -*Membrane protein insertase*	*Escherichia coli*	60 kDa 6 TM	GFPd	NP_418161
PheP -Phenylalanine-specific permease	*Escherichia coli*	50 kDa 12 TM	GFPe	NP_415108
YijD -Inner membrane protein	*Escherichia coli*	13 kDa 4 TM	GFPe	P_418399
GltP-Glutamate-aspartate symport protein	*Escherichia . coli*	47 kDa 10 TM	GFPd	ACI73820
YfbF-Undecaprenyl-phosphate 4-deoxy-4-formamido-L-arabinose transferase	*Escherichia coli*	36 kDa 2 TM	GFPe	YP_002408357
YgfU-Uric acid transporter	*Escherichia coli*	50 kDa 13 TM	GFPe	KLX82054
YqcE-Inner membrane protein	*Escherichia coli*	46 kDa 12 TM	GFPe	ANK03046
Ct6 Mot A proton channel	*Chlorbium tepidum*	46 kDa 4 TM	GFPe	Q8KC40
Dr10 MscS channel	*Deinococcus tepidum*	40 kDa 5 TM	GFPe	Q9RXU5
Dr 35 Multidrug efflux transporter	*Deinococcus tepidum*	44 kDa 12 TM	GFPe	Q9RZF0
DV1 Na/Ca exchanger family	*Desulfovibrio vulgaris*	44 kDa 12 TM	GFPe	Q723816
Ph1 CIC channel	*Pyrococcus horikoshi*	40 kDa 11 TM	GFPe	Q8U0I6
Dv12 Na/H antiporter	*Desulfovibrio vulgaris*	45 kDa 13 TM	GFPe	Q72AL4
Dv 3 MFS transporter	*Desulfovibrio vulgaris*	40 kDa 11TM	GFPe	Q72WJ3
Sp17 Formate/nitrate	*Silicibacter*	26 kDa	GFPe	Q5LW76

family transporter	*pomeroy*	10TM		
Oo3 MscS channel	*Oenococcus onei*	32 kDa<br>3 TM	GFPe	Q04DD4
Tt14 transporter	*Thermus thermophilus*	42 kDa<br>10 TM	GFPe	Q72HL7
Gs21 Ms channel superfamily	*Geobacter sulfurreducens*	60 kDa<br>6 TM	GFPe	Q74CF1
Ss21 amino acid transporter-like iron(III) ABC transporter, permease	*Sulfolobus solfataricus*	49 kDa<br>12 TM	GFPe	NP_343113.1
SQR: Sulfide:quinone oxidoreductase	human	50kDa<br>monotopic	None	Q9Y6N5

Table S3: analysis of the toxicity of the expression plasmids

Bacterial host	BL21(DE3)	C44(DE3)	C45(DE3)
Growth medium	LB	2xTY	2xTY
**Expression plasmids**	**RCSA=SA(+IPTG)/SA(-IPTG) (%)**		
pHis17 – sfGFP	0	116	40
pET28a+ - YijD-GFP	0	82	53
pET28a+ - PheP-GFP	0	102	200
pET28a+ - YgfU-GFP	0	105	57
pET28a+ - YqcE-GFP	0	62	156
pET28a+ - YidC-GFP	0	77	43
pET28a+ - GltP-GFP	0	90	143
pET28a+ - YfbF-GFP	0	143	200
pLIC - Ct6-EGFP	0	81	30
pLIC - Dr10-EGFP	0	81	70
pLIC - Dr35-EGFP	0	71	32
pLIC - Dv1-EGFP	0	41	40
pLIC - Ph1-EGFP	0	32	0
pLIC - Dv12-EGFP	0	85	67
pLIC - Dv3-EGFP	0	49	42
pLIC - Sp17-EGFP	0	40	38
pLIC - Tt14-EGFP	0	57	34
pLIC - Oo3-EGFP	0	49	45
pLic - Ss21-EGFP	0	58	11
pLic - Gs21-EGFP	0	16	0
**Bacterial host**	**C43(DE3)**	**C44(DE3)**	**C45(DE3)**
pHis17hSQR	120	126	59

Table S4: PCR primers used to confirm mutations in C44(DE3) and C45(DE3)

Name	Sequence	Bacterial mutant
Gene 1 for	GGCCCTTGAGCATGAGTCTT	C44(DE3)
Gene 1 rev	GAGACTCGTGCAACTGGTCA	C44(DE3)
rbsD for	TGATATTTCATCGGTGATCTCCC	C44(DE3)
rbsD rev	CGAATTTCAATGGTATTTCCCTG	C44(DE3)
Gene 1 for (PCR)	GGCCCTTGAGCATGAGTCTT	C45(DE3)
Gene 1 rev (PCR)	GAGACTCGTGCAACTGGTCA	C45(DE3)
Gene 1 sequencing primer	TCTGGCTTGCCTAACCAGTG	C45(DE3)
rbsD for	TGATATTTCATCGGTGATCTCCC	C45(DE3)
rbsD rev	CGAATTTCAATGGTATTTCCCTG	C45(DE3)
gltL for	CGGTGCAGCAAGGTGAAATC	C45(DE3)
gltL rev	CTGCGCCGGAAACTTATTGG	C45(DE3)
YcdX for	AGTGGTTGATGGGGTAGGGA	C45(DE3)
YcdX rev	TCGCTTGTGTATTGGTCGCT	C45(DE3)
B-lom for	GCGCGAATATGCCGGTTATC	C45(DE3)
B-lom rev	GCCACCTCTTCCACCATCAG	C45(DE3)

Table S5: Genetic changes in C41(DE3) and C43(DE3) bacterial hosts

Gene	Function	Mutation	Position from nucleotide 1 of coding sequence	Region	Effect
Present only in **C41(DE3)**					
proY	Predicted cryptic proline transporter	T → A*	438	coding	Synonymous
melB	Melibiose:sodium symporter	C → T*	653	coding	Ala → Val
ycgO	Potassium/proton antiporter	C → A*	1103	coding	Gly → Val
yhhA	Hypothetical protein	C → T*	290	coding	Pro → Leu
ydcD	Hypothetical protein	G → A	71	coding	Gly → Glu
zwf	Glucose-6-phosphate 1-dehydrogenase	C → A	815	coding	Ala -> Asp
rpoC	DNA-directed RNA polymerase subunit beta	G → T	3023	coding	Gly → Val
Present only in **C43(DE3)**					
dcuS	Sensor of fumarate two component system; frame-shifted in BL21(DE3)	GCGCC deletion*	866–870	coding	Frameshift
fur	Ferric uptake regulator	insertion*	392	coding	Val insertion
lacI	*lac* repressor	G → T*	574	coding	al → Phe
lon	DNA-binding ATP-dependent protease La	IS*4* excision*	− 156 from *lon*	intergenic	Activation of Lon protease
yibJ	Predicted Rhs-	C → A*	90	coding	Synonymous

	family protein				
yjcO	Hypothetical protein	A → G*	665	Coding	Glu → Gly
cydA	Cytochrome d terminal oxidase, subunit I	IS*1* insertion*	− 262 from *cydA*	Promoter	N.D.
ccmF ~ ompC*		IS*1* deletion*	21-kb large deletion	N.D.	
yjiV ~ yjjN*		IS*1* deletion*	37-kb large deletion	N.D.	
***Present in both* C41(DE3) *and* C43(DE3)**					
l	T7 DNA-directed RNA polymerase	AA – > GT, G – > A	P*lac*UV5 – 10 P*lac*UV5 CAP binding site	Promoter	
rbsD	Predicted cytoplasmic sugar binding protein; disrupted by insertion of IS in BL21(DE3)	IS*3* excision**	217–1586	Coding	N.D.
yehU	Predicted sensory kinase in two component system	T – > G	679	Coding	Phe → Val

*Mutation identified in the publication of Kwon and coworkers (Kwon et al. 2015); **change also identified in C44(DE3) and C45(DE3).

Table S6. Genetic changes in C44(DE3) and C45(DE3) bacterial hosts.

Gene	Function	Mutation	Chromosomal coordinate of the mutations	Effect
***Present only in* C44(DE3)**				
**T7, 1**	T7 RNA polymerase	C → T	752,380	Gln_{656}→ TAG (*ambre*)
***Present only in* C45(DE3)**				
**T7, 1**	T7 RNA polymerase	T→ G	751,489	Glu_{359}→ TAA (*ochre)*
gltL	Glutamate/aspartate ATP binding protein subunit	Deletion of insJK2 insertion	642,461-643,903	Restore Leu_{118} in frame with Lys_{119}
B-lom	DE3 Δ(B, C, nu3, D, E, Fi, Fii, Z, U, V, G, T, H, M, L, K, I, J, lom)	Deletion	771,453-788,063	from B(Leu_{49}) to lom(Phe_{206})
ycdX	Hypothetical protein	Deletion	1,101,730-1,101,744	Frameshift from Phe_{100}
***Present in both C44(DE3) and* C45(DE3)**				
rbsD	D-ribose pyranase	Deletion of insJK5 insertion	3,822,152-3,824,017	Restore Val_{64} in frame with Asp_{65}

Table S7: real time quantitative PCR primers

Name	Sequence
sfGFP f	ACCCGGATCACATGAAGCAG
sfGFP r	CAGGATGTTGCCGTCCTCTT
ihf f	CAAGACGGTTGAAGATGCAGT
ihf r	GCAAAGAGAAACTGCCGAAA
T7 RNA P f	AGTCAAGCTGGGCACTAAGG
T7 RNA P r	CACTGCGAGTAACACCGTGA

Table S8: ANOVA test values

Figure 3A				
**Plasmid**	**F-value**	**p-value**		
		BL21(DE3) / C45(DE3)	BL21(DE3)/ C44(DE3)	C45(DE3) / C44(DE3)
YidC - GFP	F (2, 6) = 166.0	P < 0.0001	P = 0.9999	P < 0.0001
PheP - GFP	F (2, 6) = 9.311	P = 0.0240	P = 0.9934	P = 0.0212
YijD - GFP	F (2, 6) = 23.55	P = 0.0013	P = 0.0080	P = 0.1956
GltP - GFP	F (2, 6) = 424.7	P < 0.0001	P < 0.0001	P < 0.0001
YfbF - GFP	F (2, 6) = 321.5	P < 0.0001	P < 0.0001	P = 0.0003
YgfU - GFP	F (2, 6) = 172.1	P < 0.0001	P < 0.0001	P = 0.0026
YqcE - GFP	F (2, 6) = 13.26	P = 0.0258	P = 0.0060	P = 0.4168

Table S8: ANOVA test values
Figure 3B

Plasmid	F-value	p-value									
		BL21(DE3) /C41(DE3)	BL21(DE3) /C43(DE3)	BL21(DE3) /C44(DE3)	BL21(DE3) /C45(DE3)	C41(DE3) /C43(DE3)	C41(DE3) /(DE3)	C41(DE3) /C45(DE3)	C43(DE3) /(DE3)	C43(DE3) /C45(DE3)	C44(DE3) /C45(DE3)
Ct6 - GFP	F (4, 22) = 10.42	0.3832	0.7888	0.0008	0.0020	0.9101	0.0129	0.0362	0.0017	0.0052	0.9897
Dr10 - GFP	F (4, 25) = 7.343	0.9899	0.9975	0.0336	0.0023	>0.9999	0.0906	0.0073	0.0679	0.0052	0.7990
Dr35 - GFP	F (4, 25) = 14.82	0.1583	0.0808	<0.0001	<0.0001	0.9968	0.0016	0.0188	0.0039	0.0411	0.8532
Dv1 - GFP	F (4, 25) = 11.10	0.1196	0.6058	<0.0001	0.0035	0.8207	0.0115	0.5383	0.0008	0.0963	0.2841
Ph1 - GFP	F (4, 22) = 1.875	0.8329	0.9948	0.1890	0.8212	0.9275	0.5640	>0.9999	0.1774	0.9182	0.5820
Dv12 - GFP	F (4, 25) = 9.746	0.9994	0.9999	0.0010	0.0092	>0.9999	0.0018	0.0159	0.0015	0.0131	0.9006
Dv3 - GFP	F (4, 25) = 9.953	0.3195	0.4955	<0.0001	0.0516	0.9975	0.0034	0.8643	0.0015	0.6960	0.0348
Sp17 - GFP	F (4, 25) = 8.689	0.9980	0.9992	0.0007	0.0532	>0.9999	0.0016	0.1004	0.0013	0.0882	0.4019
Oo3 - GFP	F (4, 22) = 8.613	0.0965	0.3155	0.0157	<0.0001	0.8344	0.9995	0.3133	0.5527	0.0102	0.2353
Tt14 - GFP	F (4, 25) = 8.370	0.1842	0.9046	0.0002	0.0276	0.6272	0.0486	0.8856	0.0017	0.1681	0.2824
Gs21 - GFP	F (4, 20) = 6.406	0.2086	0.5348	0.0873	0.0007	0.9614	0.9882	0.0934	0.7798	0.0239	0.2213
Ss21 - GFP	F (4, 20) = 6.415	0.0094	0.0038	0.6796	0.7230	0.9939	0.1439	0.1256	0.0666	0.0573	>0.9999

Table S8: ANOVA test values

Figure 6A

Minutes after induction	F-value	p-value
**0**	F (2, 12) = 67.33	P < 0.0001
**30**	F (2, 8) = 0.2827	P = 0.7610
**60**	F (2, 12) = 4.780	P = 0.0297
**120**	F (2, 11) = 84.02	P < 0.0001

Figure 6B

Minutes after induction	F-value	p-value
**0**	F (2, 14) = 152.8	P < 0.0001
**30**	F (2, 14) = 99.73	P < 0.0001
**60**	F (2, 16) = 6.312	P = 0.0095
**120**	F (2, 24) = 147.3	P < 0.0001

Figure 6C

Condition	F-value	p-value
**- IPTG**	F (3, 8) = 55.44	P < 0.0001
**+ IPTG**	F (3, 8) = 81.18	P < 0.0001

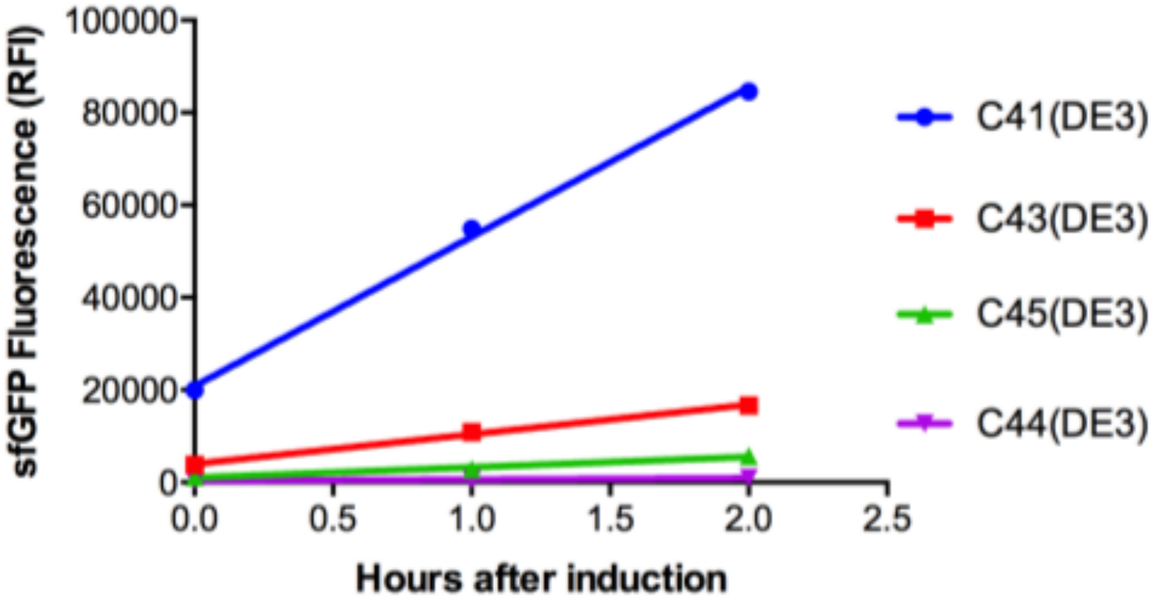

Figure S1. Analysis of sfGFP RFI in BL21(DE3) derivatives

C44(DE3) and C45(DE3) bacterial hosts were transformed with pHis17-sfGFP expression vector. 200,000 cells were counted and analyzed by flow cytometry for sfGFP relative fluorescence intensity (RFI). The graph represents the Initial rate of sfGFP fluorescence in C44(DE3) and C45(DE3) compared to C41(DE3) and C43(DE3).

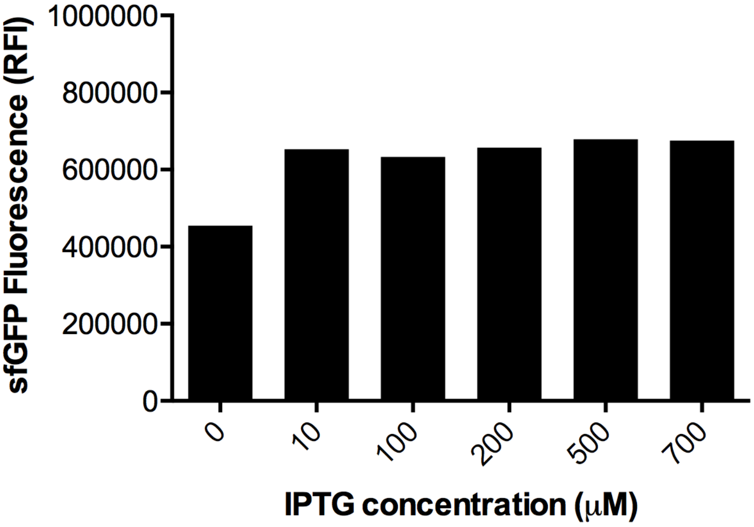

Figure S2. Regulation of the T7 expression system in BL21(DE3)

BL21(DE3) were transformed with pHis17-sfGFP expression vector. 200,000 cells were counted and analyzed for sfGFP RFI. Cells were induced at OD_{600} 0.4 with increasing concentration of IPTG. The sfGFP RFI was recorded the next day after overnight induction.

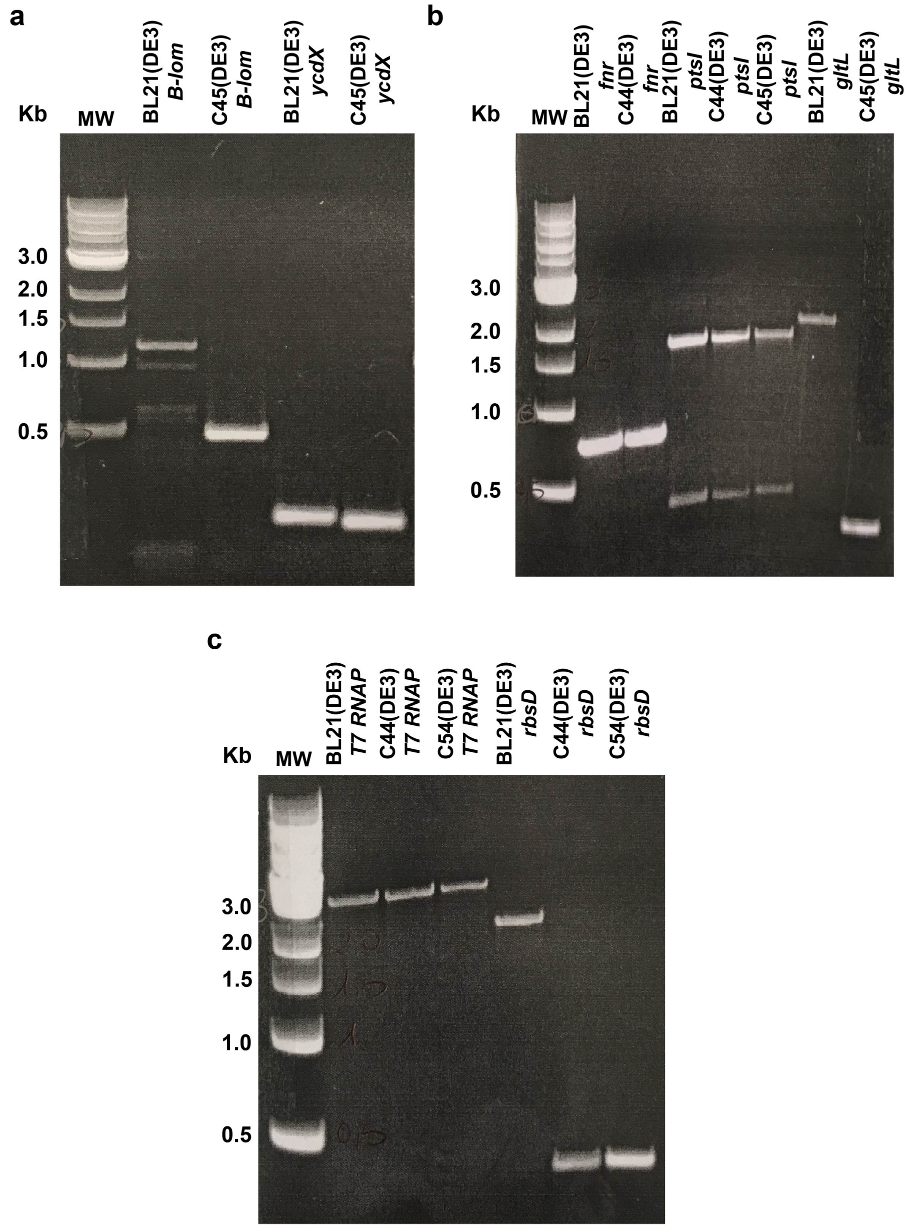

Figure S3. Confirmation of mutations in C44(DE3) and C45(DE3) bacterial hosts by PCR amplification and sequencing. (a) *B-lom* and *ycdX* C45(DE3) mutations compared to BL21(DE3) on agarose gel. **(b)** *gltL* C45(DE3) mutation compared to BL21(DE3) on agarose gel. The five lines before showing *fnr* and *ptsI* genes DNA are not of interest of this paper. **(c)** *T7 RNAP* and *rbsD* mutation in C44(DE3) and C45(DE3) compared to BL21(DE3) on agarose gel.
